# Editorial: Exploiting DNA Damage Response in the Era of Precision Oncology

**DOI:** 10.3389/fonc.2020.611127

**Published:** 2020-10-15

**Authors:** Yitzhak Zimmer, Hans Christian Reinhardt, Michaela Medová

**Affiliations:** ^1^ Department of Radiation Oncology, Inselspital, Bern University Hospital, University of Bern, Bern, Switzerland; ^2^ Department for BioMedical Research, Inselspital, Bern University Hospital, University of Bern, Bern, Switzerland; ^3^ Department of Hematology and Stem Cell Transplantation, University Hospital Essen, University of Duisburg-Essen, Essen, Germany; ^4^ German Consortium for Translational Cancer Research (DKTK), Medical Faculty, University of Duisburg-Essen, Essen, Germany

**Keywords:** DNA damage, precision oncology, synthetic lethality, DNA repair, personalized medicine

The main scope of precision oncology is providing a personally tailored cancer treatment that targets specific driver alterations identified *via* a next generation sequencing profiling of a patient tumor. The application of precision oncology is evolving dramatically and is constantly reshaping cancer treatment. The already routine implementation of trastuzumab and pertuzumab for treatment of breast cancer patients with HER2/neu amplification, imatinib in chronic myeloid leukemia and gastrointenstinal stromal tumors, dabrafenib and trametinib in *BRAF^V600E^*-mutant melanoma, erlotinib and crizotinib for non-small cell lung cancer with the *EGFR* p.L858R mutation or the *ALK/EML4* rearrangement, respectively, serve as only few examples for the proof of principle (1).

Genome stability is critical for the maintenance of cellular physiology and is persistently sustained by the complex signaling networks of cell cycle checkpoint mediators and DNA repair effectors that together constitute the DNA damage response (DDR) network, which monitors and repairs damaged DNA (2–4). A major consequence of a compromised DDR function is cellular transformation and the onset and progression of cancer. Indeed, genomic instability is recognized as a major hallmark of cancer that commonly evolves on a defective DDR function background (5).

Targeting specific DDR signaling pathways in the context of precision oncology offers opportunities on two different, but complementary levels. Firstly, the vast majority of anti-cancer conventional approaches that consist of radiation therapy, as well as chemotherapeutic drugs as for example platinum compounds, topoisomerase inhibitors and temozolomide, elicit their cytotoxicity *via* DNA damage. Deregulated upregulation of particular DDR pathways by cancer cells may provide an escape mechanism that results in more efficient DNA repair with consequent treatment resistance and less favorable prognosis (6). Depending on a particular tumor landscape, a personalized targeted intervention within a specific relevant DDR pathway may therefore be instrumental for overcoming treatment resistance *via* chemo-radiosensitization. In that respect, effective and specific targeting of the three DDR master upstream kinases of the PIKK family, ATM, ATR and DNA-PK, is in the center of major research efforts in the last years (7–9). An additional targeting concept to effectively induce tumor cell death is blocking of cell cycle checkpoint mediators, such as CHK1, CHK2, and WEE1 in cancer cells with a high replication stress, allowing therefore cell cycle progression with a high burden of DNA damage (10).

The second major venue to utilize DDR targeting in personalized cancer treatment are tumors with loss-of-function mutations in genes encoding DDR components of a particular repair pathway. These mutations may create an ultimate dependency on an alternative pathway, which, if targetable, creates a tumor-specific vulnerability in the form of a synthetic lethal interaction exploitable in the clinic. Obviously, the dogma of targeting synthetic lethal interactions in the context of DDR signaling has been established through the integration of PARP inhibitors in the management of homologous recombination-deficient tumors due to *BRCA1/BRCA2* inactivating mutations (11–15). Motivated by this successful clinical implementation, functional genomic screens are profoundly used to identify novel synthetic interactions and drug targets in human cancers (16).

This Research Topic of Frontiers in Oncology entitled “Exploiting DNA Damage Response in the Era of Precision Oncology” aimed at bringing together contributions covering various aspects of DDR targeting in the context of precision oncology frameworks. The scopes of the research and review articles included in this collection are described below:

- Mohiuddin and Kang discuss in their review the biologic rationale for DNA-PK as a target in cancer. The roles of DNA-PK within the DDR, as well as in non-DDR signaling are described and an updated overview over the pharmacological efforts for generating effective inhibitors is provided.
- In the original research article by Lundgren Mortensen et al. the authors explore a tumor radiosensitization approach, which aims at increasing cellular p53 levels by using a stapled peptide, PM2, that interferes with the MDM2/X-dependent p53 downregulation. The effectiveness of PM2 together with external beam radiotherapy has been investigated in a panel of cancer cells.
- Das et al. have presented data of a computational approach to predict synthetic lethal interactions of somatic mutations in DDR genes within a TCGA-based pan-cancer cohort of patients. They have used various *in silico* approaches, including drug sensitivities responses, to validate the novel described synthetic lethal interactions.
- Carr et al. investigated in their research on acute myeloid leukemia a combination of the novel DNA-PK inhibitor M3814 and Mylotarg, an approved CD33 antibody conjugated with the DNA double strand break-inducing drug calicheamicin. The study demonstrated an enhanced anti-tumor activity of Mylotarg in the combined treatment modality, resulting from the inhibition of the DNA double strand break repair through non-homologous end-joining *via* M3814.
- Baldwin et al. explored the efficacy of a nano-formulation of the PARP inhibitor talazoparib in combination with temozolomide in xenograft models of Ewing Sarcoma. Their data suggest that the nanoparticle formulation of talazoparib reduces the toxicity of the combined treatment as compared to oral administration of the PARP inhibitor.
- Meng et al. reviewed the signaling interplay between DDR pathways, RNA processing and the generation of tumor-associated extracellular vesicles that are linked to treatment resistance and metastasis.
- The minireview by Trenner and Sartori focused on most recent updates concerning DNA double strand breaks repair pathways and how they could be exploited further for cancer treatment. Particular emphasis of this work is on combinatorial therapeutic approaches and the targeting of potentially newly discovered synthetic lethal interactions.
- Liptay et al. reviewed mechanisms of acquired drug resistance in tumors with DDR deficiencies. The authors of this study concentrated primarily on BRCA-deficient cancers and the emerging role of replication fork biology in acquired drug resistance in these tumors.
- Burgess et al. reviewed mechanisms involved in genomic instability of lung tumors and therapeutic opportunities in combination of DDR-based targeting with various modalities including immunotherapies.

Our understanding of the intricate and extremely complex network of the cellular DDR reshaped by groundbreaking discoveries in the last decades allowed numerous successful implementations of these findings into clinical practice. At the same time, the more we know, the more new questions arise. To one of them – who will profit from a specific therapy? –precision oncology will have to furnish answers all over again and again.

## Author Contributions

All authors contributed equally. All authors contributed to the article and approved the submitted version.

## Conflict of Interest

The authors declare that the research was conducted in the absence of any commercial or financial relationships that could be construed as a potential conflict of interest.
